# 3D models from EOS imaging to assess axial changes in the lumbar spine after selective thoracic fusion in adolescent idiopathic scoliosis (AIS)

**DOI:** 10.1007/s00590-026-04696-z

**Published:** 2026-03-07

**Authors:** Matthew Bellamy, Raveen Jayasuriya, Shreya Srinivas, Michael Athanassacopoulos, Edward Bayley, Lee Breakwell, Ashley Cole

**Affiliations:** 1 The Medical School, University of Sheffield, Sheffield, UK; 2 Department of Paediatric Orthopaedics and Spinal Surgery, Sheffield Children’s NHS Foundation Trust, Sheffield, UK

**Keywords:** EOS imaging, 3-dimensional reconstruction, Selective thoracic fusion, Axial plane, Lumbar spine rotation, Pelvic rotation

## Abstract

**Purpose:**

Instrumented scoliosis correction to T12/L1 can be a full correction (FC) of a single thoracic curve or a selective thoracic fusion (STF) where there is a significant lumbar curve. This study aims to evaluate the utility and feasibility of 3-dimensional (3D) EOS modelling to quantify pre- and post-operative axial plane changes in the uninstrumented lumbar spine.

**Methods:**

This study included patients undergoing primary surgery (2018–2021) for AIS Lenke 1 or 3, with the lowest instrumented level at T12-L1 and reconstructable EOS bi-planar images available pre-op, post-op, and at 1-year follow-up. EOS 3D modelling gives the apical rotation and mean rotation form T1-L5 from a neutral pelvis.

**Results:**

Twenty patients (age 14.3; 7 Lenke 1 A (FC), 13 Lenke 1B–3 C (STF)) were included. Lumbar Cobb correction averaged 54% (1 A), 41% (1B), and 21% (1 C/3 C). Neither the STF nor FC achieved notable correction of apical lumbar rotation (1 A: −5%, *p* > 0.05, 1B; +0°, *p* > 0.05, 1 C/3 C; +2.5°, *p* > 0.05). Uninstrumented average L1-L5 rotation showed no significant change at 1 year for any curves. A significant correlation was observed between lateral bending Cobb angles and L1-L5 average rotation at one year (*p* < 0.05). Notably, EOS imaging measured greater axial rotation than PA x-rays, with differences normalising after accounting for pelvic parameters.

**Conclusion:**

EOS 3D modelling is valuable for visualising the mobile lumbar spine. Our models showed no significant correction of lumbar rotation, and a large impact from pelvic rotation on radiographic measurements. Increased curve flexibility may improve axial correction. The apparent reduction in lumbar rotation on plain radiographs is more likely attributable to pelvic rotation.

## Introduction

Instrumented scoliosis correction of just the structural thoracic curve in the presence of a compensatory lumbar curve (Lenke 1B, 2B, 2C, 3C) is termed a ‘selective thoracic fusion’ (STF) [1]. STF aims to minimise the vertebral levels of the fusion surgery to maintain flexibility in the lumbar spine, important for patients to remain active [1]. However, if only the main thoracic curve is instrumented, the lumbar curve can fail to correct spontaneously, and extension of fusion may be required [2]. The Lenke classification uses a Cobb angle of 25 degrees or more on side bending x-rays to determine if the curve is structural or not [3]. If the lumbar curve is structural (> 25 degrees), the Lenke classification suggests that the curve should be included in the instrumentation and fusion [3]. However, since the classification was produced, it has been shown that patients with Lenke 3C curves can be treated successfully by only instrumenting the main thoracic curve [3].

While STF improves coronal curve parameters, studies often overlook other planes [4]. Previous work demonstrated that although STF achieved 50% Cobb correction in 30 patients, their lumbar translation and axial rotation was unchanged or deteriorated [5]. Furthermore, these rotation measurements used plain radiographs rather than contemporary 3D modelling [6].

In 2018, Jankowski et al. used the EOS imaging system to review 55 AIS cases with major thoracic or thoracolumbar/lumbar curves. By measuring axial rotations from 3D models, they found that lumbar rotation improved from 22 (+/- 7) degrees to 11 (+/- 5 degrees). However, these results represented full posterior corrections, and no sub-analysis was performed for selective fusion patients with an uninstrumented, mobile lumbar spine [7]. Pasha et al. isolated curve types for selective fusion by examining 21 Lenke 1B or 1 C cases undergoing “true” STF. Although not the primary outcome, their analysis of EOS scans revealed no significant difference in pre-operative versus 2-year post-operative lumbar axial rotation [8] .

Alongside coronal Cobb correction, de-rotation is important for surgical success, as it reduces the rib and lumbar prominences that may contribute to cosmetic deformity [9]. Due to the linked nature of the thoracic and lumbar spine deformity, changes to the thoracic spine will likely be translated into the lumbar spine [3]. While previous literature demonstrates effective coronal Cobb correction in both the thoracic and lumbar spine, axial plane results remain rare, as traditional measurement methods are frequently inaccurate and time-consuming [10]. Computed tomography (CT) scans have been utilised to assess the spontaneous correction of the axial plane after STF, showing promising early results [11]. However, CT scans are taken supine which changes the 3D spinal deformity compared to weight bearing images [12]. The 3D EOS modelling enables weight-bearing assessment of post-operative correction in uninstrumented lumbar vertebrae, quantifying axial plane changes [6]. Furthermore, EOS imaging takes its reference from a neutral pelvis, showing normalised and standardised axial rotation [6] .

This pilot study evaluated the utility and feasibility of 3D EOS modelling for assessing pre- and post-operative axial alignment, specifically quantifying changes in the mobile lumbar spine and pelvis following selective thoracic fusion.

## Methods

**Fig. 1:**
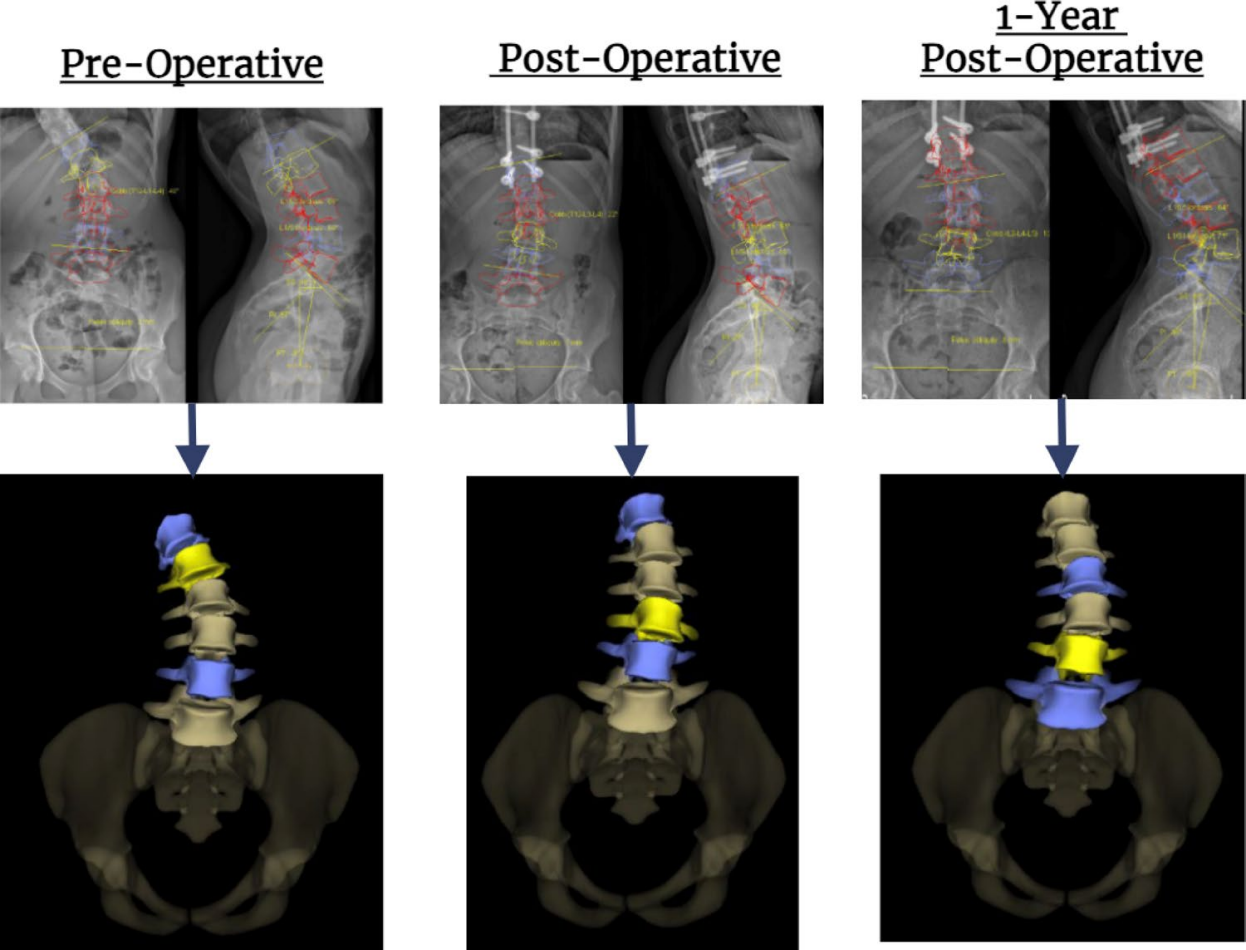
3D reconstructions of the lumbar spine in all 3 planes at differing clinical timepoints

This retrospective cohort study included all patients with a diagnosis of adolescent idiopathic scoliosis, instrumentation ending no more caudal than L1 and bi-planar EOS imaging pre-operatively, post-operatively and at a minimum of one year follow up (Fig. 1). Exclusion criteria were patients with non-idiopathic aetiology, only posteroanterior EOS images, EOS images without the acetabulum or pelvic parameters visible or patients with less than one year follow up. The development and implementation of this study was approved by the NHS Health.

Research Authority and by the Sheffield Children’s Hospital Research & Development department (HRA:4632). The 3D spinal measurements were measured on the sterEOS software, accessed through EOS imaging.

Apical vertebral rotation was treated as an absolute value with lowest instrumented vertebra (LIV) to LIV + 4 rotations including negative values. Further data gathered from the 2D radiographs included: Lenke classification, stable vertebra (CSVL bisected vertebra), lowest instrumented vertebra (LIV), apical vertebra rotation (AVR, measured using Cerny et al. method [13]) and the thoracic Cobb angle. Lumbar Cobb angle on bending was also measured. EOS bi-planar imaging was taken in a weightbearing position (full or micro dose). The full spine protocol was set to model from the LIV to L5 with no blinding. Radiographs included the femoral heads and acetabulum to allow for rotational measurements. Only EOS images containing bi-planar, pelvic measurements were deemed ‘reconstructable’, as these are required to calculate full rotational values from T1 through L5.

All cases were reviewed by four spinal surgeons during a monthly planning meeting. When a full correction was planned, the LIV was always positioned at the last touching stable vertebra. In cases where an under correction was planned, the LIV was selected to bisect the stable vertebra. During surgery, under corrections involved reduced implant density, more flexible rods, and deliberate under-rotation of the thoracic curves. Additionally, the LIV was left slightly tilted in the coronal plane to accommodate the planned under correction.

Statistical analysis was performed on SPSS v28.0 (IBM, 2023). Statistical significance was accepted at a p value less than 0.05. Statistical analysis was conducted across the whole population and across different Lenke classifications. This created 3 distinct groups: Lenke 1A, 1B and 1C/3 classifications. Lenke 1C and 3C classifications were grouped as they both have large lumbar curves with large apical translation and associated rotation.

Spearman’s rank was used to measure non-parametric correlations. Mann-Whitney U tests were used for paired variables. Wilcoxon Rank analysis was used to determine the association between non-parametric paired variables. Normality was checked with a Shapiro-Wilk test with a *p*-value of < 0.05 indicating non-parametric data.

## Results

**Fig. 2:**
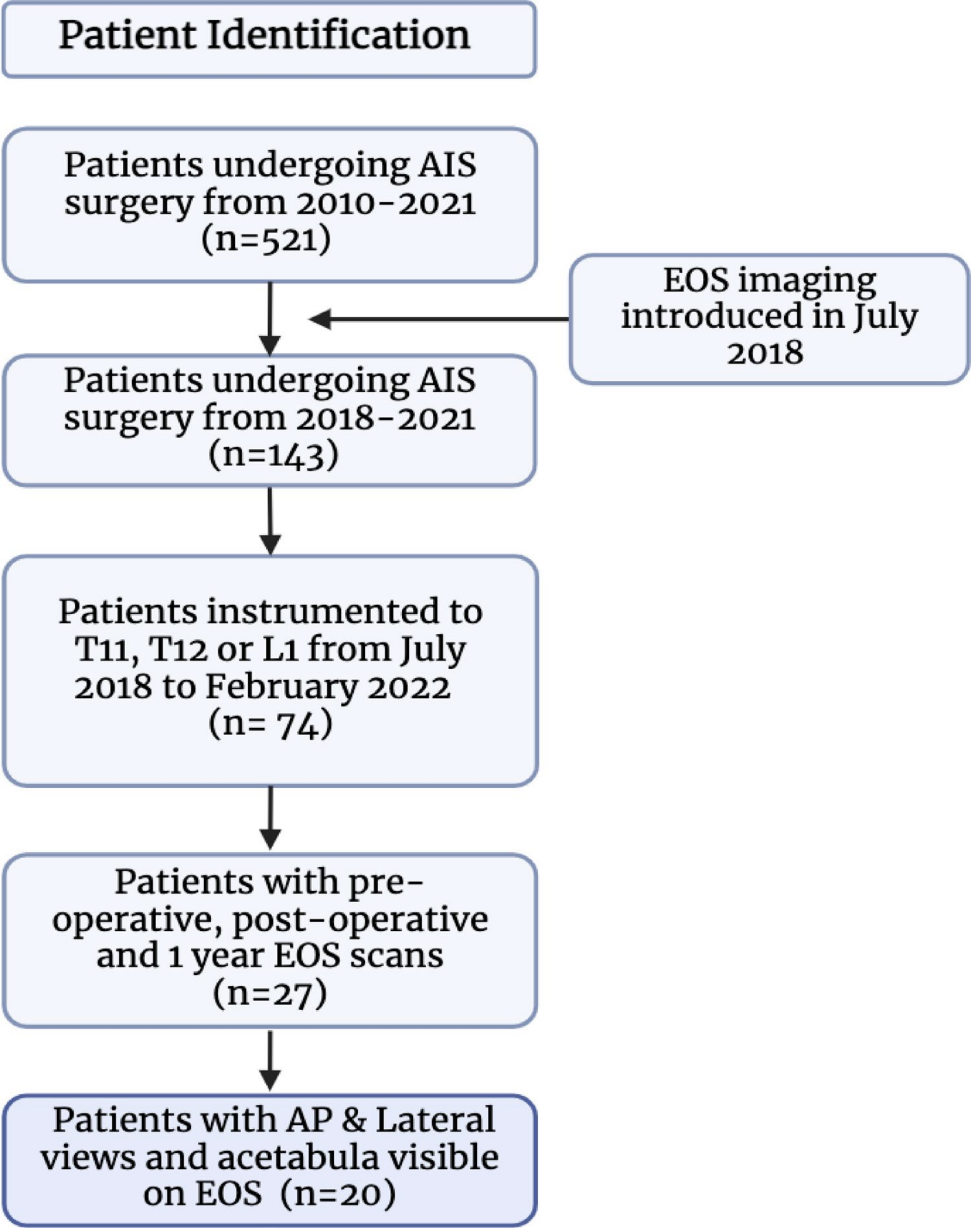
Patient inclusion criteria flow diagram

Twenty patients met the inclusion criteria (Fig. 2). Average age of the patients at the time of surgery was 14.3 years. Average 1-year post-operative follow up with bi-planar EOS x-ray was 15.1 months. The distribution of curve types showed that 7, 9 and 4 patients had Lenke 1 A, 1B and 1 C/3 curves respectively (Table 1). Surgical technique variation meant that 12 patients underwent convex correction, and 8 patients had concave corrections.

**Table 1:** Demographic summary table of the whole cohort and Lenke classifications

	All Cases	1A	1B	1C/3
	*N* = 20	*N* = 7	*N* = 9	*N* = 4
Age at fusion surgery (years)*	14.3 (1.2)	14.7 (0.7)	14.1 (1.0)	14.3 (2.1)
Follow up time (months) *	15.1 (5.3)	14.6 (4.9)	16.9 (6.2)	11.9 (1.2)
Concave correction §	8 (40.0)	4 (57.1)	4 (44.4)	4 (100)
Convex correction §	12 (60)	3 (42.9)	5 (55.6)	0
Lowest instrumented vertebrae §				
T11	1 (5)	0	0	1
T12	7 (35)	2	4	2
L1	12 (60)	5	5	1
Type of correction §				
Full	17 (85)	7 (100)	8 (89)	2 (50)
Under	3 (15)	0	0	2 (50)
Thoracic Cobb pre-operatively (2D x-ray)	56.6	60.4	55.5	52.5
Thoracic Cobb 1-Year (2D x-ray)	28.9	29.8	28.1	29.5
Lumbar Cobb pre-operatively (EOS)*	40.6 (6.1)	39 (8.1)	41.1 (4.0)	42.3 (7.1)
Lumbar Cobb bending (2D x-ray) *	14.5 (11.5)	11.8 (11.8)	12.6 (7.2)	23.25 (17.2)
Lumbar Cobb angle at 1 year (EOS) *	23.7 (10.8)	17.9 (7.2)	24 (11.1)	33.3 (10.3)

* Mean (Standard Deviation)

§ Number (%) of Patients

### Curve correction

The thoracic Cobb angle measured on 2-dimensional (2D) PA x-ray was significantly corrected by 27.7 degrees (48.8%; SD: 9.7; *p* < 0.001) across the whole cohort. Lumbar Cobb angle measured on 3D EOS was corrected by 16.9 degrees (41.6%; SD: 10.5; *p* < 0.001). The L1-L5 and L1-S1 lordosis was not significantly changed with a difference of 1.2 degrees (SD: 8.6; *p* = 0.555) and 1.3 degrees (SD: 9.0; *p* = 0.528) respectively. There were no significant changes across any other pelvic parameters (*p* > 0.05).

In the transverse plane, the lowest instrumented vertebra (3.7 degrees; *p* = 0.007), lowest instrumented vertebra + 1 level (4.2 degrees; *p* = 0.037) and lowest instrumented vertebra + 4 levels (4.0 degrees; *p* = 0.014) were all significantly changed on 3D EOS from the pre-operative to the 1 year follow up. The rotation in the apical vertebra and the rotation averaged across the lumbar curve did not significantly change from pre-operatively to 1 year when analysed across the whole cohort. For Lenke 1 A curves, a maximal correction of the thoracic curve was performed in surgery with attempted segmental derotation. For Lenke 1B and C curves, a more limited frontal plane correction was performed by using a 6 mm titanium rod (more flexible than cobalt chrome) and allowed the rod to rotate slightly into the coronal plane to retain more thoracic scoliosis and with no formal derotation manoeuvre.

### Apical and average lumbar rotation analysed by Lenke classifications

Lenke 1 A patients had a lower starting lumbar apical rotation than both Lenke 1Bs and Lenke 1Cs & 3s. The apical vertebral rotation was corrected by 38.5% in Lenke 1A patients, 0% in Lenke 1B patients and worsened by 14.7% in Lenke 1C/3 patients (Table 2). Across Lenke 1A, 1B, and 1C/3 classifications, the average L1-L5 lumbar rotation decreased between the post-operative and 1-year timepoints. Lenke 1 A patients experienced the greatest average rotational correction, despite having the smallest initial rotation magnitude (Table 2).

**Table 2:** 3D EOS (relative to the pelvis) measured axial rotation changes across differing Lenke classifications

Lenke classification	Pre-operative lumbar AVR	1 Year lumbar AVR	Median change	% Change
1A	13.0	8.0	−5.0	−38.5
1B	17.0	17.0	0	0
1C/3	17.0	19.5	2.5	+ 14.7

Lenke classification	Pre-operative ALR	1 Year ALR	Median change	% Change
1A	9.0	7.0	−2.0	−22.2
1B	12.5	11.5	−1.0	−8.0
1C/3	15.0	14.3	−0.7	−5.0

AVR, Apical vertebral rotation; ALR, Average lumbar rotation

### Comparison of 3D EOS and 2D X-ray pelvic measurements

**Fig. 3:**
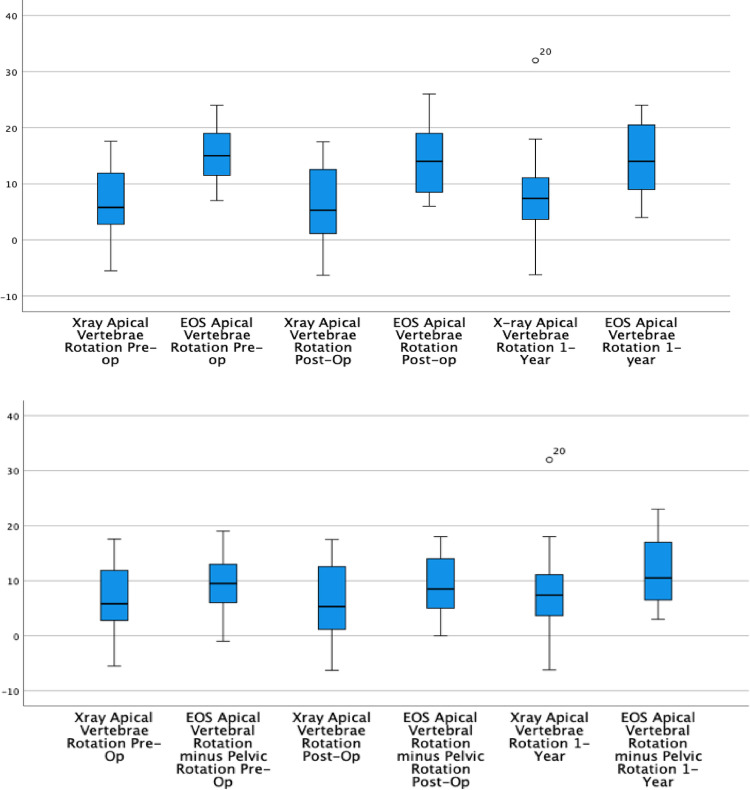
Box-plot showing the difference between mean axial rotations measured on EOS imaging and PA x-ray (top) and EOS imaging without pelvic rotation and PA x-ray (bottom)

Pelvic rotation on EOS was assessed by differences between the acetabula on the lateral EOS x-ray. Table 3 demonstrates that the rotational measurements of lumbar apical vertebra taken from 2D x-ray are more likely to be smaller than the rotations measured by EOS 3D models. When the axial rotation measured at the pelvis is subtracted from the apical rotation derived from EOS imaging, the resulting values demonstrate greater agreement in magnitude with the axial rotation measured on 2D x-ray. Figure 3 shows that accounting for pelvic rotation at the postoperative assessment significantly normalises the discrepancy observed between the 2D x-ray and EOS measurements of apical vertebral rotation.

**Table 3:** EOS imaging and PA 2D x-ray measured axial rotation values with and without the pelvic rotation included in EOS values

	Mean EOS rotation	Mean 2D x-ray rotation	Mean difference	SD	Significance
Pre-op apical rotation*	15.5	7	−8.5	3.5	< 0.001
Pre-op apical rotation (no pelvic rotation)	9.8	7	−2.8	9.4	0.196
Post-op apical rotation*	14.4	5.7	−8.7	5.6	< 0.001
Post-op apical rotation (no pelvic rotation)	9.1	5.7	−3.4	9.4	0.126
1 year apical rotation*	14.5	8.2	−6.3	5.9	< 0.001
1 year apical rotation (no pelvic rotation)	11.5	8.2	−3.3	10.3	0.172

*Relative to pelvic rotation

### Lenke classification, curve flexibility and apical correction

**Fig. 4:**
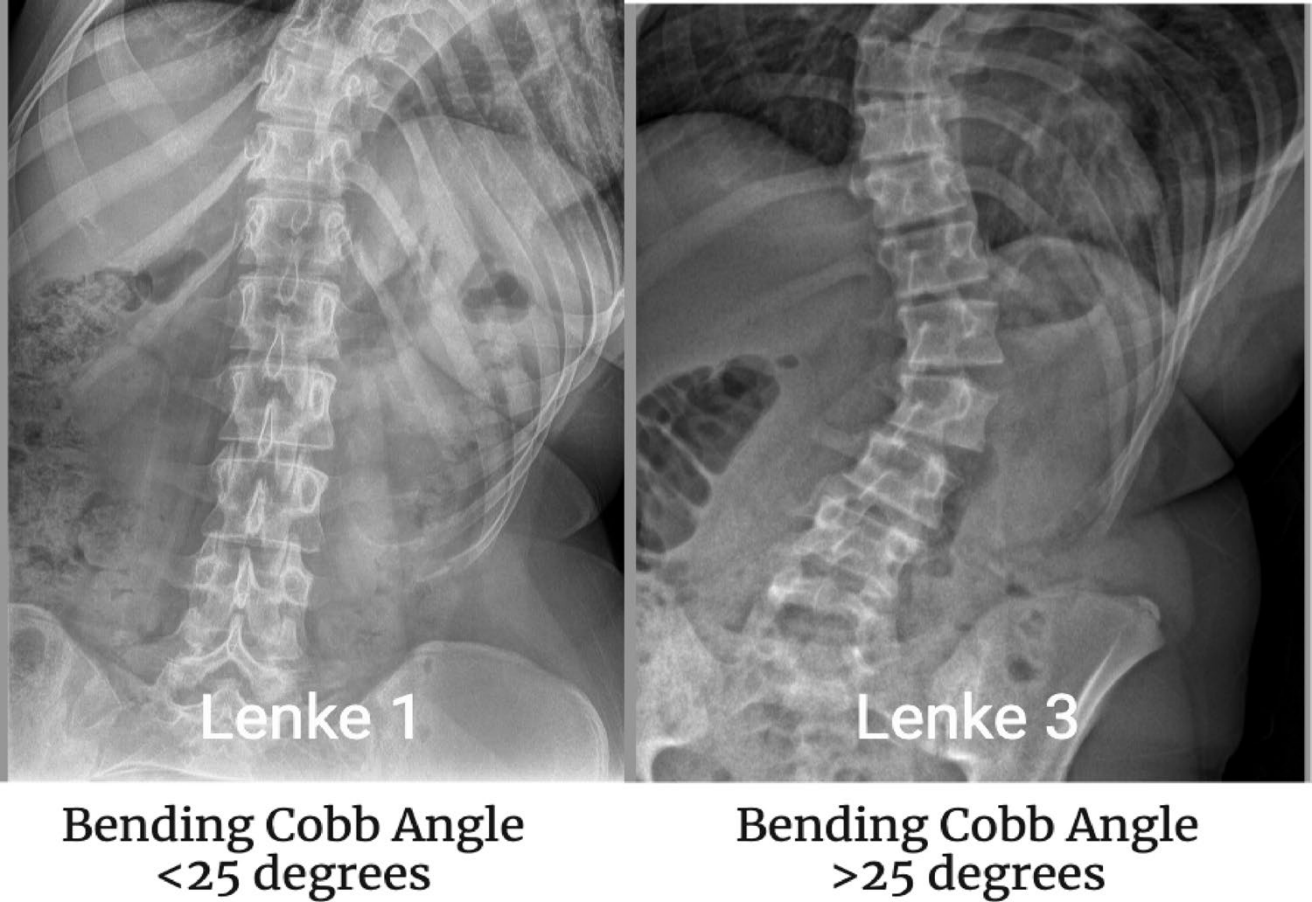
The difference in Cobb angle correction on bending between Lenke 1 curves and Lenke 3 curves

**Fig. 5:**
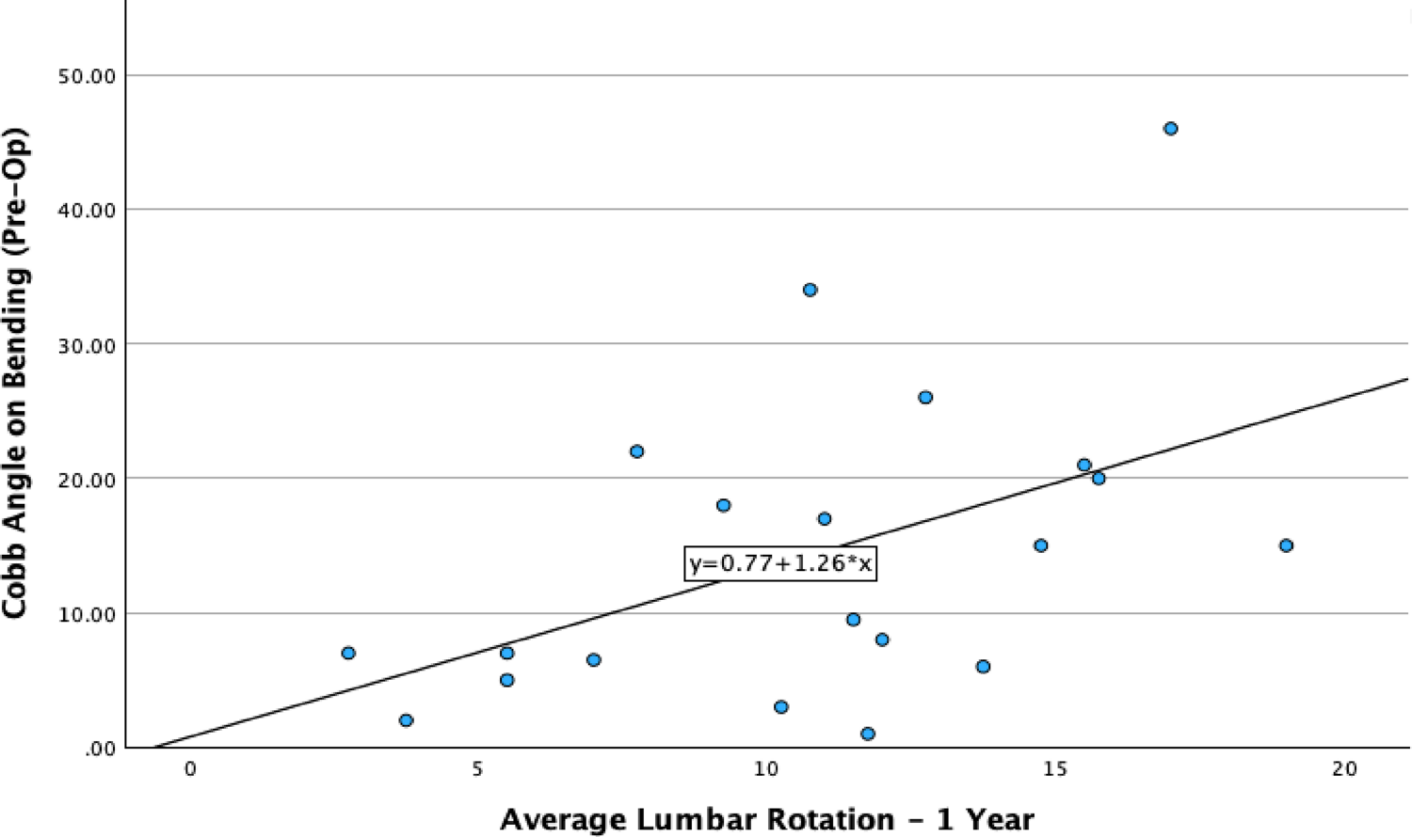
Correlation between the Cobb angle achieved on bending and the average lumbar rotation at 1 year post-operatively

The most significant difference between Lenke groups that achieved a good lumbar rotational correction and those that did not was the preoperative bending angle. The Lenke 1A group demonstrated the largest bending correction, which can be attributed to its inherent flexibility compared to the stiffer Lenke 3 group. The group that corrected their lumbar apical rotation had an average Cobb angle on bending of 7 degrees, while the worsening group had a Cobb angle on bending of 21 degrees (Fig. 4). Spearman’s correlation shows a significant correlation between bending angle and L1-L5 average rotation at 1 year (*P* < 0.05) (Fig. 5).

### Complications

There were no surgical complications or cases of coronal imbalance in thispatient cohort. Additionally, no patients required revision surgery at the one-year follow-up.

## Discussion

EOS 3D model provides meaningful and helpful clinical data to monitor the mobile uninstrumented lumbar spine after selective thoracic fusion. Visualisation of all three planes of the spine from a rotationally neutral pelvis has previously not been possible without supine CT or MRI scans. Because of this, traditional methods of reporting success after these surgeries have often been isolated to the coronal plane. More recent publications have started to demonstrate the importance of axial and sagittal plane parameters in the success of these operations [8–15].

This study shows no significant change to the apical or average lumbar rotation across the whole cohort. This is different to what a 2017 CT based study found in their cohort of 30 patients who reported a mean correction of 32% across the apical rotation [11]. Sub-analysis of our patient cohort by Lenke classification revealed significant differences in apical vertebral rotation. Lenke 1A patients demonstrated nearly 40% correction, whereas Lenke 1B patients showed no change. In contrast, Lenke 1C/3 patients experienced a worsening of nearly 15%. This discrepancy may be due to the increased stiffness of the large secondary curve distal to the surgical instrumentation. A correlation was also observed between the preoperative Cobb angle on bending and the average L1-L5 rotation at one year. Specifically, a larger preoperative bending Cobb angle was associated with a greater L1-L5 rotation. The Lenke 1C/3 group had a baseline bending Cobb of around 24 degrees, compared to the 12 degrees for the more flexible 1A group.

The increased stiffness in the Lenke 1C/3 group is also reflected in the difference between their one-year postoperative Cobb angle and their preoperative bending Cobb angle. The average one-year Cobb angle for the more flexible Lenke 1A group was only 6 degrees greater than their bending Cobb, while the Lenke 1C/3 group had a one-year Cobb angle that was, on average, over 10 degrees greater than their preoperative bending Cobb. The use of pre-operative bending x-rays and the link between apical vertebral rotation is gaining importance in modern literature [16]. The use of 3D EOS models to evaluate postoperative patient outcomes in all three planes of the spine is a novel approach with the potential to predict which patients will respond favourably to STF.

Comparing these results to a recent study using 3D EOS models to assess the timing of changes in the lumbar spine after selective thoracic fusion, we see similar results [8]. In their results they found a 56% reduction in their lumbar Cobb, a 17% reduction in lumbar lordosis and a 30% worsening of the apical vertebral rotation at 1 year reducing to 20% at 2 years [8]. A 1996 study found similar results to the one conducted 20 years later. Although curves with a King classification 2, 3 or 4 had an average Cobb correction on 53.1%, 46.9% and 58.3% respectively, worsening rotation was seen in the lumbar spine of both King type 2 and 3 curves [17]. Spinal rotation is often reversed in the lumbar spine compared to the thoracic and with large, inflexible curves the residual “stiffness” in the system may translate the de-rotation of the thoracic spine into the lumbar curve [4].

EOS imaging has the advantage of providing a fast, global axial view of essential spinal imaging which can be used for monitoring and the prediction of progression in patients after STF [18]. Recent studies are highlighting the need to account for pelvic rotation before surgery to understand the full extent of spinal imbalance [19]. Measurements taken from the radio-plane without accounting for pelvic rotation can mean the sagittal profile as well as the axial rotations are significantly different to those taken with pelvic axial rotation accounted for [19]. We found that traditional x-ray methods often underrepresent the true extent of the spinal rotation [20]. This is likely due to the principal that EOS 3D spinal models will measure rotation from an axially neutral pelvis. This will often add more rotation onto the rotation visible on plain x-ray as the pelvis is often rotated in the opposing direction to the lumbar vertebra. This study highlights the necessity to consider pelvic parameters in assessing lumbar correction after selective thoracic fusion. EOS imaging provides these automated global clinical parameters as standard with 3D model from an axially balanced patient plane [6]. The clinical significance of pelvic rotation is unknown, but because it often mirrors the rotation of the unfused lumbar spine, it can underrepresent spinal deformity on PA radiographs. Using 3D reconstructions overcomes this by showing the true lumbar rotation against a neutral pelvic baseline.

The increased rotation in Lenke 1/3C curves means that the surgeon must consider the merits of a selective thoracic fusion over a fusion of both curves. To maintain a mobile lumbar curve there may need to be sacrifices at surgery in potentially limiting or possibly increasing the rotation in the instrumented thoracic spine to prevent transmission to the lumbar spine. Further work is required to see whether the post-operative pelvic rotation in the same direction as the thoracic spine and opposite direction of the lumbar spine may represent a compensatory mechanism to maintain ‘rotational balance’.

EOS imaging with 3D reformatting adds additional rotational information in STF when the lumbar curve has a Lenke C modifier, and possibly Lenke B. This feedback to surgeons is needed to refine and improve selective thoracic fusion surgery in these young patients and potentially the limits of surgery beyond which we should be fusing both curves. For Lenke 1A thoracic curves, PA and lateral radiographs seem adequate for post-operative assessment, particularly considering cost and accessibility of EOS imaging.

There are limitations that are important to mention in this study. Due to the recent introduction of EOS imaging to our department, we could only include patients with a one-year follow-up. Furthermore, only a small subset of this population had scans that extended sufficiently to include the pelvis to measure rotation which may introduce selection bias. This study also relies on anatomical landmark mapping using bi-planar x-rays to create 3D models. The 3D models that are produced are prone to larger measurement error unlike 3D models created from CT scanning. We also have a relatively small sample size due to the necessity to have both PA and lateral views alongside imaging extending down to the pelvis. The heterogeneous nature of the curve patterns and lack of control group potentially dilute the conclusions of the study. Although patient posture on image acquisition has standard guidelines in our centre, EOS imaging is prone to large variations in standing position for patients with scoliosis. This change in positioning could lead to variations in pelvic and vertebral rotation alignment. We did not assess shoulder balance either in the frontal or axial planes and the effect that this might have on the correction of the lumbar curve. This is needed in future research.

## Conclusion

3D models from EOS imaging have the potential to provide useful clinical information from all 3 spinal planes both pre and post operatively. The rotational measurements in the lumbar spine differ significantly when measured on EOS 3D models compared to 2D x-ray due the addition of pelvic rotation in EOS models. At 1-year postoperatively, coronal correction in the thoracic and lumbar spine is significantly decreased with small changes to the sagittal and pelvic parameters. In Lenke 1A curves (full correction) the apical vertebrae de-rotates by 37.5%. In Lenke 1B curves there is no change in rotation while Lenke 1C/3 curves have a 14.7% increase in apical rotation despite surgical under-correction of the instrumented thoracic curve. A lower lumbar Cobb angle on pre-operative bending x-ray and therefore more flexible lumbar spine is more likely to have a better rotational correction at 1 year.

## Data Availability

The data and analysis that supports the results of this study are available from the corresponding author, M Bellamy, upon reasonable request.

## Abbreviations

3D: 3-dimensional
2D: 2-dimensional
STF: Selective thoracic fusion
AVR: Apical vertebral rotation
ALR: Average lumbar rotation
PA: Posteroanterior
LIV: Lowest instrumented vertebra
CT: Computed tomography
MRI: Magnetic resonance imaging
L1-L5: Lumbar 1 to lumbar 5 vertebra
CSVL: Central sacral vertical line

## References

[1] Fischer CR, Kim Y (2011) Selective fusion for adolescent idiopathic scoliosis: a review of current operative strategy. Eur Spine J 20:1048. 10.1007/S00586-011-1730-9 21387194 10.1007/s00586-011-1730-9 PMC3176697

[2] Prablek M, McGinnis J, Winocour SJ, Reece EM, Kakarla UK, Raber M et al (2021) Failures in thoracic spinal fusions and their management. Semin Plast Surg 35:20. 10.1055/S-0041-1723832 33994874 10.1055/s-0041-1723832 PMC8110356

[3] Lenke LG, Edwards CC, Bridwell KH (2003) The Lenke classification of adolescent idiopathic scoliosis: how it organizes curve patterns as a template to perform selective fusions of the spine. Spine (Phila Pa 1976):28. 10.1097/01.BRS.0000092216.16155.33 10.1097/01.BRS.0000092216.16155.33 14560193

[4] Lenke LG, Betz RR, Bridwell KH, Harms J, Clements DH, Lowe TG (1999) Spontaneous lumbar curve coronal correction after selective anterior or posterior thoracic fusion in adolescent idiopathic scoliosis. Spine (Phila Pa 1976) 24:1663–1672. 10.1097/00007632-199908150-00007 10472100 10.1097/00007632-199908150-00007

[5] Peelle MW, Boachie-Adjei O, Charles G, Kanazawa Y, Mesfin A (2008) Lumbar curve response to selective thoracic fusion in adult idiopathic scoliosis. Spine J 8:897–903. 10.1016/J.SPINEE.2007.11.010 18261962 10.1016/j.spinee.2007.11.010

[6] Garg B, Mehta N, Bansal T, Malhotra R (2020) EOS^®^ imaging: concept and current applications in spinal disorders. J Clin Orthop Trauma 11:786. 10.1016/J.JCOT.2020.06.012 32879565 10.1016/j.jcot.2020.06.012 PMC7452333

[7] Jankowski PP, Yaszay B, Cidambi KR, Bartley CE, Bastrom TP, Newton PO (2018) The relationship between apical vertebral rotation and truncal rotation in adolescent idiopathic scoliosis using 3D eeconstructions. Spine Deformity 6:213–219. 10.1016/J.JSPD.2017.10.003/METRICS 29735128 10.1016/j.jspd.2017.10.003

[8] Pasha S, Flynn JM, Sponseller PD, Orlando G, Newton PO, Cahill PJ (2017) Timing of changes in three-dimensional spinal parameters after selective thoracic fusion in Lenke 1 adolescent idiopathic scoliosis: two-year follow-up. Spine Deformity 5:409–415. 10.1016/J.JSPD.2017.04.003 29050718 10.1016/j.jspd.2017.04.003

[9] Hwang SW, Samdani AF, Lonner B, Miyanji F, Stanton P, Marks MC et al (2012) Impact of direct vertebral body derotation on rib prominence: are preoperative factors predictive of changes in rib prominence? Spine (Phila Pa 1976):37. 10.1097/BRS.0B013E31821FD379 10.1097/BRS.0b013e31821fd379 21540768

[10] Scaramuzzo L, Giudici F, Bongetta D, Caboni E, Minoia L, Zagra A (2017) Thoraco-lumbar selective fusion in adolescent idiopathic scoliosis with Lenke C modifier curves: clinical and radiographic analysis at 10-year follow-up. Eur Spine J 26:514–523. 10.1007/S00586-017-5152-1 28547576 10.1007/s00586-017-5152-1

[11] Demura S, Murakami H, Kato S, Yoshioka K, Yonezawa N, Takahashi N et al (2017) Spontaneous derotation of compensatory lumbar curve after thoracic fusion in adolescent idiopathic scoliosis. Spine Surg Relat Res 1:27–30. 10.22603/SSRR.1.2016-0006 31440609 10.22603/ssrr.1.2016-0006 PMC6698538

[12] Mahato NK, Maharaj P, Clark BC (2024) Lumbar spine anatomy in supine versus weight-bearing magnetic resonance imaging: detecting significant positional changes and testing reliability of quantification. Asian Spine J 18:1. 10.31616/ASJ.2023.0203 38287663 10.31616/asj.2023.0203 PMC10910142

[13] Cerny P, Marik I, Pallova I (2014) The radiographic method for evaluation of axial vertebral rotation - presentation of the new method. Scoliosis 9:1–9. 10.1186/1748-7161-9-11/FIGURES/8 25120581 10.1186/1748-7161-9-11 PMC4130697

[14] Mladenov KV, Vaeterlein C, Stuecker R (2011) Selective posterior thoracic fusion by means of direct vertebral derotation in adolescent idiopathic scoliosis: Effects on the sagittal alignment. Eur Spine J 20:1114–1117. 10.1007/S00586-011-1740-7/FIGURES/1 21380744 10.1007/s00586-011-1740-7 PMC3176688

[15] Pasha S, Cahill PJ, Flynn JM, Sponseller P, Newton PO (2018) Relationships between the axial derotation of the lower instrumented vertebra and uninstrumented lumbar curve correction: radiographic outcome in Lenke 1 adolescent idiopathic scoliosis with a minimum 2-year follow-up. J Pediatr Orthop 38:e194–201. 10.1097/BPO.0000000000001136 29360660 10.1097/BPO.0000000000001136

[16] Kaya O, Kara D, Gok H, Kahraman S, Sanlı T, Karadereler S et al (2022) The importance of lumbar curve flexibility and apical vertebral rotation for the prediction of spontaneous lumbar curve correction in selective thoracic fusion for lenke type 1 and 2 C curves: retrospective cohort study with a mean follow-up of more than 10 years. Global Spine J 12:1516. 10.1177/21925682221098667 35485204 10.1177/21925682221098667 PMC9393973

[17] Hosman AJF, Slot GH, Van Limbeek J, Beijneveld WJ (1996) Rip hump correction and rotation of the lumbar spine after selective thoracic fusion. Eur Spine J 5:394–399. 10.1007/BF00301967/METRICS 8988382 10.1007/BF00301967

[18] Illés TS, Lavaste F, Dubousset JF (2019) The third dimension of scoliosis: the forgotten axial plane. Orthop Traumatology: Surg Res 105:351–359. 10.1016/J.OTSR.2018.10.021 10.1016/j.otsr.2018.10.021 30665877

[19] Zuckerman SL, Cerpa M, Sardar ZM, Lenke LG (2021) Don’t forget the pelvis: accounting for pelvic rotation in the preoperative assessment of adolescent idiopathic scoliosis. J Spine Surg 7:181. 10.21037/JSS-20-675 34296030 10.21037/jss-20-675 PMC8261564

[20] Lam GC, Hill DL, Le LH, Raso JV, Lou EH (2008) Vertebral rotation measurement: a summary and comparison of common radiographic and CT methods. Scoliosis 3:16. 10.1186/1748-7161-3-16 18976498 10.1186/1748-7161-3-16 PMC2587463

